# WGS-based telomere length analysis in Dutch family trios implicates stronger maternal inheritance and a role for *RRM1* gene

**DOI:** 10.1038/s41598-019-55109-7

**Published:** 2019-12-10

**Authors:** Lilit Nersisyan, Maria Nikoghosyan, The Genome of the Netherlands consortium, Laurent C. Francioli, Androniki Menelaou, Sara L. Pulit, Clara C. Elbers, Wigard P. Kloosterman, Jessica van Setten, Isaäc J. Nijman, Ivo Renkens, Paul I. W. de Bakker, Freerk van Dijk, Pieter B. T. Neerincx, Patrick Deelen, Alexandros Kanterakis, Martijn Dijkstra, Heorhiy Byelas, K. Joeri van der Velde, Mathieu Platteel, Morris A. Swertz, Cisca Wijmenga, Pier Francesco Palamara, Itsik Pe’er, Kai Ye, Kai Ye, Eric-Wubbo Lameijer, Matthijs H. Moed, Marian Beekman, Anton J. M. de Craen, H. Eka D. Suchiman, P. Eline Slagboom, Victor Guryev, Abdel Abdellaoui, Jouke Jan Hottenga, Mathijs Kattenberg, Gonneke Willemsen, Dorret I. Boomsma, Elisabeth M. van Leeuwen, Lennart C. Karssen, Najaf Amin, Fernando Rivadeneira, Aaron Isaacs, Albert Hofman, André G. Uitterlinden, Cornelia M. van Duijn, Mannis van Oven, Manfred Kayser, Martijn Vermaat, Jeroen F. J. Laros, Johan T. den Dunnen, David van Enckevort, Hailiang Mei, Mingkun Li, Mark Stoneking, Barbera D. C. van Schaik, Jan Bot, Tobias Marschall, Alexander Schönhuth, Jayne Y. Hehir-Kwa, Robert E. Handsaker, Paz Polak, Mashaal Sohail, Dana Vuzman, Karol Estrada, Steven A. McCarroll, Shamil R. Sunyaev, Fereydoun Hormozdiari, Vyacheslav Koval, Carolina Medina-Gomez, Ben Oostra, Jan H. Veldink, Leonard H. van den Berg, Steven J. Pitts, Shobha Potluri, Purnima Sundar, David R. Cox, Peter de Knijff, Qibin Li, Yingrui Li, Yuanping Du, Ruoyan Chen, Hongzhi Cao, Jun Wang, Ning Li, Sujie Cao, Jasper A. Bovenberg, Gert-Jan B. van Ommen, Arsen Arakelyan

**Affiliations:** 1 Bioinformatics Group, Institute of Molecular Biology NAS RA, 7 Hasratyan str., 0014 Yerevan, Armenia; 2 Institute of Biomedicine and Pharmacy, Russian-Armenian University, 123 Hovsep Emin St, 0051 Yerevan, Armenia; 3 Department of Medical Genetics, Center for Molecular Medicine, University Medical Center Utrecht, Utrecht, The Netherlands; 4 Department of Genetics, University Medical Center Groningen, University of Groningen, Groningen, The Netherlands; 5 Genomics Coordination Center, University Medical Center Groningen, University of Groningen, Groningen, The Netherlands; 6 Department of Computer Science, Columbia University, New York, New York USA; 7 The Genome Institute, Washington University, St. Louis, Missouri USA; 8 Section of Molecular Epidemiology, Department of Medical Statistics and Bioinformatics, Leiden University Medical Center, Leiden, The Netherlands; 9 European Research Institute for the Biology of Ageing, , University Medical Center Groningen, University of Groningen, Groningen, The Netherlands; 10 Department of Biological Psychology, VU University Amsterdam, Amsterdam, The Netherlands; 11 Department of Epidemiology, Erasmus MC University Medical Center Rotterdam, Rotterdam, The Netherlands; 12 Department of Forensic Molecular Biology, Erasmus MC University Medical Center Rotterdam, Rotterdam, The Netherlands; 13 Leiden Genome Technology Center, Department of Human Genetics, Leiden University Medical Center, Leiden, The Netherlands; 14 Netherlands Bioinformatics Center, Nijmegen, The Netherlands; 15 Department of Evolutionary Genetics, Max Planck Institute for Evolutionary Anthropology, Leipzig, Germany; 16 Bioinformatics Laboratory, Department of Clinical Epidemiology, Biostatistics and Bioinformatics, Academic Medical Center, Amsterdam, The Netherlands; 17 SURFsara, Science Park, Amsterdam, The Netherlands; 18 Centrum Wiskunde & Informatica, Life Sciences Group, Amsterdam, The Netherlands; 19 Department of Human Genetics, Radboud University Nijmegen Medical Center, Nijmegen, The Netherlands; 20 Center for Neuroscience, Donders Institute for Brain, Cognition and Behaviour, Radboud University Nijmegen Medical Center, Nijmegen, The Netherlands; 21 Program in Medical and Population Genetics, Broad Institute of Harvard and MIT, Cambridge, Massachusetts USA; 22 Department of Genetics, Harvard Medical School, Boston, Massachusetts USA; 23 Division of Genetics, Department of Medicine, Brigham and Women’s Hospital, Harvard Medical School, Boston, Massachusetts USA; 24 Department of Genome Sciences, University of Washington, Seattle, Washington USA; 25 Department of Internal Medicine, Erasmus MC University Medical Center Rotterdam, Rotterdam, The Netherlands; 26 Analytic and Translational Genetics Unit, Department of Medicine, Massachusetts General Hospital, Boston, Massachusetts USA; 27 Department of Clinical Genetics, Erasmus MC University Medical Center Rotterdam, Rotterdam, The Netherlands; 28 Department of Neurology, Brain Center Rudolf Magnus, University Medical Center Utrecht, Utrecht, The Netherlands; 29 Rinat-Pfizer, Inc., South San Francisco, California, USA; 30 Department of Clinical Genetics, Leiden University Medical Center, Leiden, The Netherlands; 31 Forensic Laboratory for DNA Research, Department of Human Genetics, Leiden University Medical Center, Leiden, The Netherlands; 32 BGI-Shenzhen, Shenzhen, China; 33 BGI-Europe, Copenhagen, Denmark; 34 Department of Biology, University of Copenhagen, Copenhagen, Denmark; 35 The Novo Nordisk Foundation Center for Basic Metabolic Research, University of Copenhagen, Copenhagen, Denmark; 36 Legal Pathways Institute for Health and Bio Law, Aerdenhout, The Netherlands; 37 Department of Systems Biology, Columbia University, New York, New York USA; 38 Department of Human Genetics, Leiden University Medical Center, Leiden, The Netherlands; 39 Department of Epidemiology, Julius Center for Health Sciences and Primary Care, University Medical Center Utrecht, Utrecht, The Netherlands

**Keywords:** Genome informatics, Functional genomics

## Abstract

Telomere length (TL) regulation is an important factor in ageing, reproduction and cancer development. Genetic, hereditary and environmental factors regulating TL are currently widely investigated, however, their relative contribution to TL variability is still understudied. We have used whole genome sequencing data of 250 family trios from the Genome of the Netherlands project to perform computational measurement of TL and a series of regression and genome-wide association analyses to reveal TL inheritance patterns and associated genetic factors. Our results confirm that TL is a largely heritable trait, primarily with mother’s, and, to a lesser extent, with father’s TL having the strongest influence on the offspring. In this cohort, mother’s, but not father’s age at conception was positively linked to offspring TL. Age-related TL attrition of 40 bp/year had relatively small influence on TL variability. Finally, we have identified TL-associated variations in ribonuclease reductase catalytic subunit M1 (*RRM1* gene), which is known to regulate telomere maintenance in yeast. We also highlight the importance of multivariate approach and the limitations of existing tools for the analysis of TL as a polygenic heritable quantitative trait.

## Introduction

The terminal regions of linear human chromosomes are composed of telomeres - sequences of tandem TTAGGG repeats normally stretching around 10–15 kb in length. They protect the chromosomes from degradation and end-to-end fusions^1^ and perform a number of regulatory functions, including regulation of gene expression^2^, DNA damage response^3^, modulation of cellular senescence^4^, proper chromosome anchoring to the nuclear membrane and segregation during meiosis^5^. Accordingly, changes in telomere length (TL) have been linked to development of age-related diseases and cancers^6–9^.

TL regulation is a complex phenomenon that depends on a number of variables, such as age^10^, genetic^11,12^ and environmental^13,14^ factors, and the tissue under study^15,16^. Initially, the idea of telomere length dependence on chronological age was based on the fact that in most somatic cells, telomeres get shorter over an individual’s lifespan, mostly due to telomere end-resection that occurs after each round of DNA replication^17,18^. However, while telomere attrition is associated with cellular senescence, it’s not completely clear how TL is linked to organismal ageing^19^. Particularly, inverse correlation of TL with age has been shown to be true for some^20,21^, but not for all populations^22,23^. Our previous study on South Asian genomes has identified no relationship with age in this population^23^, confirming previous observations suggesting that age-dependency of telomere length might be population-specific^22^.

Genetic factors influencing TL have been identified by multiple genome-wide association studies, both in healthy and in diseased populations^11,12^. Single nucleotide variations (SNVs) in several loci, such as telomerase components *TERT* and *TERC*, and others (*OBFC1, RTEL1, etc*.) have been linked to TL^11,24,25^. For many loci, however, the identified associations with telomere length are either irreproducible in other cohorts or show population-specificity^26^. Additionally, a number of environmental factors, such as stress, exposure to oxidative agents, smoking, alcohol consumption and lifestyle, all affect telomere attrition rate^13,14^.

Finally, while telomere length regulation and environmental factors affect TL variability in a lifetime of a single individual, TL variability between individuals is also largely explained by inheritance of parental telomeres and variable TLs at birth, with a stronger link between offspring and either father’s^27^, or mother’s^28^ TL. Additionally, paternal (PAC) and maternal (MAC) ages at conception also affect TL in the offspring^29^. This has mostly been shown for PAC, and explained by relatively longer TL in the sperm of older males^27,30,31^. A recent study has simulated this effect across many generations and has suggested this as a mechanism for adaptation to cancer^32^.

Overall, based on their importance in healthy ageing and in regulation of basic cellular functionality, telomeres are being extensively studied for their role in development of age-related diseases, cancers and reproduction^6,10,24,25^. Involvement of multiple genetic and environmental factors and heritability of TL largely complicates investigation of TL regulation and necessitates multi-factor analysis. We have previously studied TL in the South Asian population, based on whole genome sequencing (WGS) data. In this population, we have identified TL-associated loci in the *ADARB2* gene, but did not reveal relation of TL with age^23^. That dataset, however, did not allow for analysis of TL inheritance patterns. Here, we make use of whole-blood derived DNA sequencing data generated by the Genome of the Netherlands (GoNL) project that involves WGS and SNV datasets from 250 families, comprising trios of a mother, a father and a child. Importantly, this dataset was annotated not only for ages of individuals at the time of data collection, but also for parental ages at conception^33^. We have measured mean telomere length (MTL) from WGS data using Computel^34^, and have performed a number of regression analyses to identify age association and inheritance patterns of MTL in the Dutch population, and to reveal MTL-associated genomic loci.

## Results

### Population stratification

Principal component analysis on SNVs was previously performed by the GoNL consortium^33^. They had identified a subtle substructure of the population according to geographical origin of the individuals (along the north-south axis of the Netherlands). However, there were no distinct clusters and the first and the second principal components explained very little variation in the genotype data (~0.25% and ~0.24%, respectively). Therefore, we have performed downstream association analyses with the assumption of a homogenous population.

### Regression analysis

**Figure 1:**
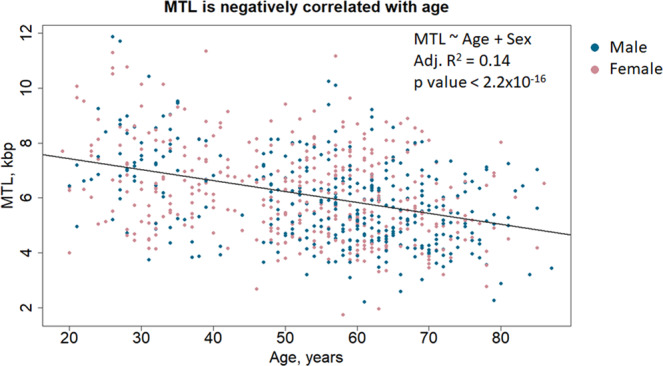
Negative association of telomere length with age. Combined analysis of children and parents shows ~40 bp reduction in MTL per year. Sex was not significantly associated with MTL after adjustment for age. Overall, age explains only 14% of MTL variability among individuals.

In the studied population, MTL ranged from as low as 1.7 to 12 kbp in length. Multiple linear regression (MLR) analysis (with the model *MTL* ~*Age* + *Sex*) on all study subjects, including the MTL of parents and children, has shown that age is negatively correlated with MTL (adjusted R^2^ = 0.14, p value < 2.2e-16), with an estimated attrition rate of ~ 40 bp per year, in agreement with prior observations^10,35^ (Fig. 1). On the other hand, contrary to studies commonly reporting longer telomeres in adult women compared to men^36^, in our cohort sex was not a significant predictor of MTL. Even though females on average had longer telomeres in our population (5.9 ± 1.6 kb in females versus 5.7 ± 1.6 kb in males, t-test p value = 0.03), this is likely due to age differences, as women were generally younger than men (by 4 years, t test p value < 0.001). Indeed, this difference was gone after adjustment for age by testing the mean difference in MTLs between males and females in each age group (females on average had 200 bp longer MTL, but with t-test p value 0.24).

Next, we have performed a series of MLRs to analyze the inheritance patterns of maternal and paternal telomeres, using MTL of children as an outcome variable and MTL of parents as predictors. For each child in our dataset, we had information regarding their age and MTL, maternal MTL (mMTL), paternal MTL (fMTL), as well as mother’s and father’s ages at conception (MAC and PAC). Pairwise correlation analysis for all these predictors (Supplementary Fig. S1) have shown that many variables were not independent, with mother’s and father’s, as well as with parents’ and child’s ages showing strong correlation, and MTL, mMTL and fMTL being correlated with each other. In order to avoid collinearity related biases, we have constructed and tested simple and complex models including one to many predictors, as well as interaction terms. Among the models with highest adjusted R^2^ value, were the simple model *MTL* ~*Age* + *mMTL* + *fMTL* + *MAC* + *PAC* (adj. R^2^ = 0.442, Supplementary Table S1), and two complex models including interaction terms. As the goodness of fit and predictors’ correlation coefficients were quite close to each other among these models, we have chosen the simple one for interpretability (see Supplementary Table S1). Exploration of regression estimates for each predictor shows that age is correlated with a scaled estimate of −0.34 when it’s the only predictor in the model. However, its influence gets three times weaker after inclusion of mother’s and father’s MTLs, which are stronger predictors of child’s MTL (single-model estimates 0.58 and 0.51, respectively).

**Figure 2:**
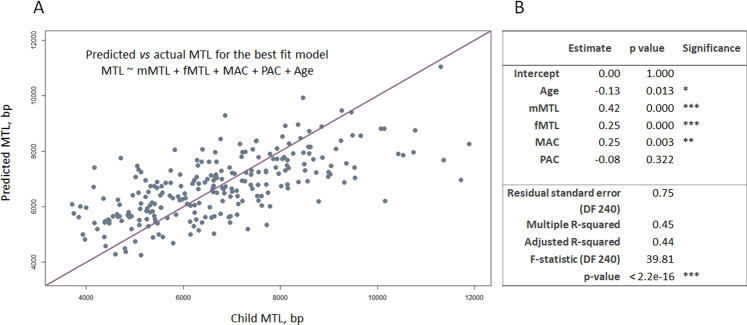
Multiple linear regression estimates for the best fit model of telomere length inheritance. (**A**) The actual MTL length of the offspring and the MTL predicted with the best fit model of telomere length inheritance: MTL ~ Age + mMTL + fMTL + MAC + Age. (**B**) The correlation estimates, and p values for MTL with offspring age, maternal and paternal MTL (mMTL, fMTL) and maternal and paternal ages at conception (MAC, PAC) are presented, along with general statistics for the test (bottom). Significant correlations are marked with one, two and three asterisks, for p values of < 0.05, <0.01 and <0.001, respectively. Note, that PAC was not a significant predictor in the model: its estimates are presented for the reader’s information.

The final model explained only 44% of the variability in the offspring MTLs (Fig. 2A), with the strongest correlate being mother’s MTL (correlation estimate = 0.42), and to a lesser extent – father’s (correlation estimate = 0.25). In this model, age was still negatively correlated with MTL, however with smaller regression estimate of only −0.13 (Fig. 2B). Interestingly, MAC was significantly and positively correlated with child’s MTL (0.25), while PAC was not a significant predictor. The observed association between MAC and child’s MTL still preserved after accounting for the rest of the variables with partial correlation analysis (partial correlation estimates for the association with offspring MTL after adjustment for the rest of the factors were −0.16 (Age, p < 0.05), 0.42 (mMTL, p < 0.001), 0.26 (fMTL, p < 0.001), 0.19 (MAC, p < 0.01) and −0.06 (PAC, p > 0.05).

### Telomere length qTL analysis

**Figure 3:**
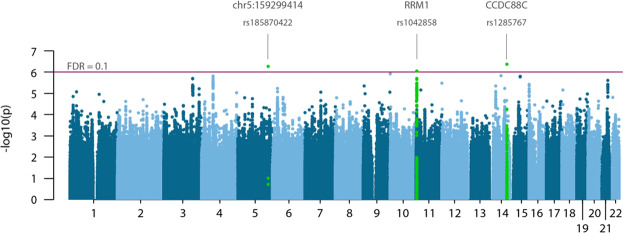
Manhattan plot for significance of SNV association with MTL. Analysis were performed with Plink family based association tests, with additive model for SNVs. Three SNVs (rs185870422 – chromosome 5q, rs1042858 – RRM1, rs1285767 – CCDC88C) are significantly associated with MTL at false discovery rate of 0.1.

Family based quantitative trait association analyses with MTL were performed with Plink and Merlin. The lists of identified top SNVs are available in Supplementary Datasheet S1. Plink revealed three SNVs significantly associated with telomere length (FDR < 0.1): rs1285767 (*CCDC88C*), rs185870422 (non-coding region on chromosome 5) and rs1042858 (*RRM1*) (Fig. 3). The current version of Plink (version 1.9) did not allow for inclusion of additional covariates, namely age, in the family based MTL association analysis. Thus, we have also performed family association tests with Merlin to obtain independent assessments of qTL. Merlin did account for age as a covariate, however, did not account for mendelian heritability of MTL. Thus, no SNVs were identified as significantly associated with MTL with this test. This is in accordance with MTL inheritance patterns revealed by MLR analysis described above, as telomere inheritance factors, particularly mother’s and father’s MTL and MAC (correlation coefficients 0.42, 0.25 and 0.25), are stronger predictors of MTL than age (correlation coefficient −0.13).

**Figure 4:**
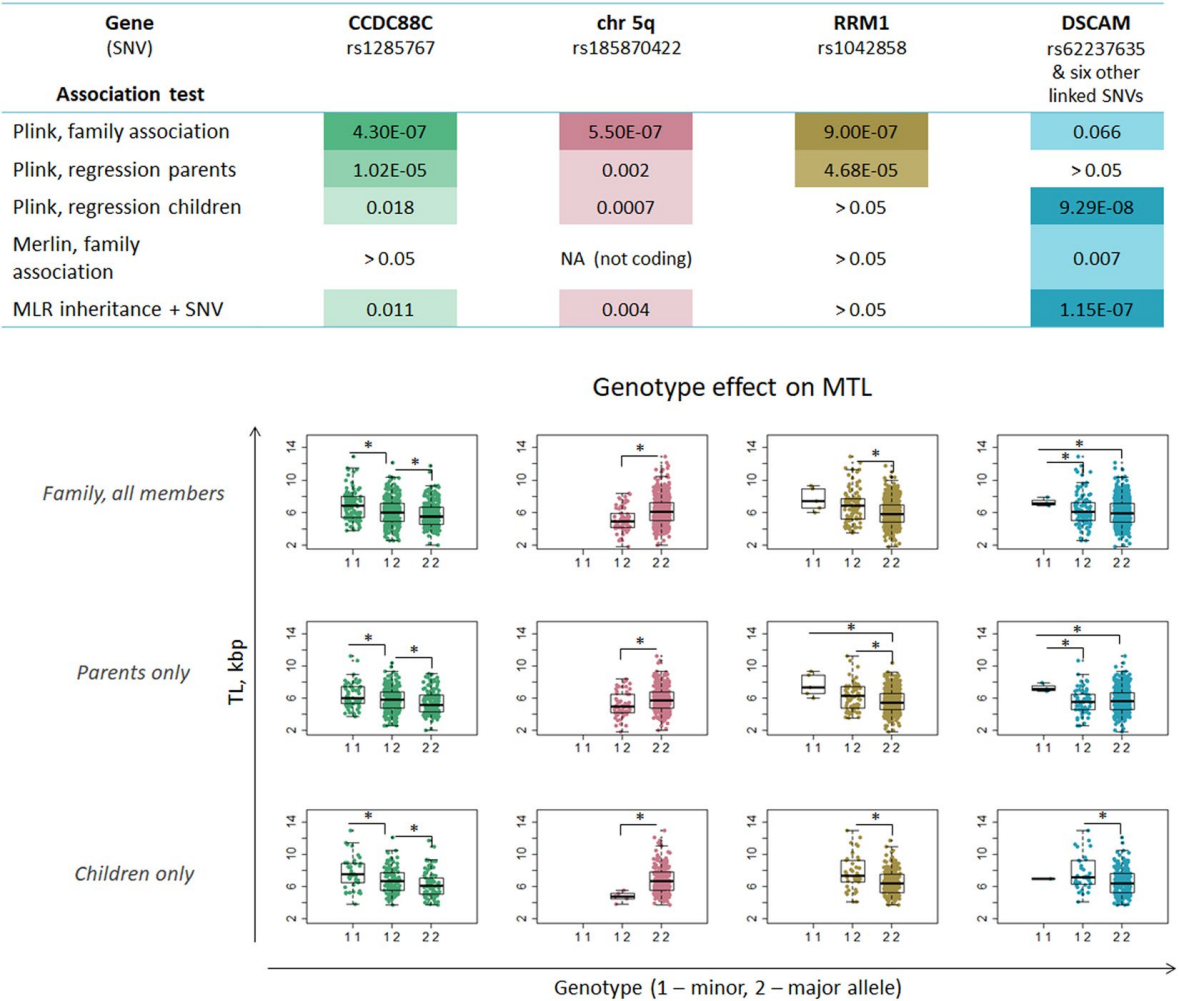
Significance and genotype effects of MTL-associated SNVs. Colors are used to delineate each SNV. The dark-to-light color gradient indicates low-to-high p values (raw p values from each test). The value of 1 × 10^−7^ (E-07) corresponds to FDR threshold of 0.1. Family tests with Merlin only accounted for coding regions, therefore no p-value for the SNV at chr5q was available for this test. The DSCAM gene was identified with six SNVs (rs11701674, rs28412810, rs11700591, rs62237637, rs11702319), which, however, were linked and showed similar patterns represented here with rs11701674 only. Boxplots show MTL values for each individual (in all the samples, or in children or parent only), depending on the amount of minor alleles for each SNV in the genotype. Significant MTL differences between genotypes are highlighted with asterisks. All the SNVs show consistent effect in parents and in children, with the minor allele of SNV at chr5q having negative, while that of the other three SNVs having positive association with MTL.

Taking into consideration the limitations of these two methods, we have also performed multiple linear regression tests for each SNV, by adding child’s genotype for each SNV to the MLR model obtained above: *MTL ~Age* + *mMTL* + *fMTL* + *MAC* + *PAC* + *SNV*. Here each SNV takes on values from 0 to 2, depending on the number of dominant alleles in a child’s genotype. These tests revealed only six SNVs residing within the *DSCAM* gene, associated with offspring MTL after adjustment for all the variables included in the MLR model. With this model, only two top SNVs from Plink association tests had significant association p values (unadjusted) (rs185870422 (chr5q) and rs1285767 (*CCDC88C*)). The results for these two SNVs and *DSCAM* were further confirmed by Plink linear regression test performed on the population of children only. Isolated Plink linear regression tests on the parents’ population did not reveal any significant quantitative loci after multiple comparison test adjustment (Fig. 4, Supplementary Datasheet S1).

One of the three loci identified by Plink, rs1285767, resides within the *CCDC88C* gene and leads to 514 bp gain of MTL per additional minor allele. *CCDC88C* encodes the protein Daple that is known to regulate the non-canonical Wnt signaling pathway, which plays an important role in embryonic development, cell motility and tumor progression^37,38^. Wnt signaling pathway is a known regulator of telomerase activity through its central player β-catenin, which binds transcription factors and regulates expression of *TERT*, the catalytic subunit of telomerase^39^. Nonetheless, the SNV is positioned intronically and its functional effects are unknown. This locus came out as a significant MTL-associated hit not only in Plink family association analysis, but was also border-line significant in separate Plink regression analyses on the populations of parents and children. The presence of minor alleles shows consistent additive effects on MTL in family and parent/children groups (Fig. 4).

The next locus identified by Plink-based family tests, as well as by Plink linear regression analysis on parent/children groups was rs185870422, residing in a non-coding region on the q arm of chromosome 5. Interestingly, no individual had a homozygous genotype for the minor allele of this SNV. However, the heterozygotes had consistently lower MTL (by 1000 bp) than homozygotes for the major allele (Fig. 4). This locus distantly interacts with *UBLCP1* and *RNF145* genes within the same chromatin loop (source: database 3DSNP version 1.0). Those, however, do not have known roles in telomere length regulation.

The *RRM1* locus was the most consistent hit in Plink family tests, with 75 SNVs having border-line significance (raw p value < 10^−6^–10^−5^), in contrast to CCDC88C, which had only two SNVs with raw p values < 10^−5^ (Fig. 4, Supplementary Datasheet S1). The rs1042858 variant of *RRM1* had positive effect on MTL (892 bp per locus), but, it was only identified during Plink family association tests and separate regression tests on parents (Fig. 4). Homozygotes for the minor allele were missing among children, but the presence of the minor allele in heterozygotes had the same positive effect on MTL. According to Ensembl Variant Effect Predictor results, this variant is synonymous to the standard transcript of *RRM1*, but can, however, introduce a modification at the 3′ UTR of its alternative transcript (ENST00000532170.5) that may affect nonsense-mediated decay.

The six SNVs revealed by the MLR model tests (rs11701674, rs28412810, rs11700591, rs62237637, rs11702319) were all linked and resided within intronic regions of the Down Syndrome cell adhesion molecule (*DSCAM*) gene), which encodes an immunoglobulin superfamily member, is involved in axon guidance and is a candidate for Down syndrome and congenital heart disease^40^. The amount of homozygotes for the minor alleles of these SNVs was low, and their effect on TL was only seen in children. In the MLR inheritance model, the regression coefficient for these SNVs was higher (0.67) compared to all other variables (mMTL had a correlation coefficient of 0.42 after addition of these SNVs). However, addition of these SNVs to the MLR model increased the percentage of explained variation in offspring MTL by only 6% (leading to regression R^2^ of 50%, Supplementary Datasheet S1). Note that while this analysis did account for all the available covariates, it didn’t account for family structure and, thus, had low statistical power.

## Discussion

Telomeres are attractive targets for aging, cancer research and reproductive medicine^6–9^. Since their discovery, a large number of studies have addressed the biology of their inheritance^27–29^ and the causes of inter-individual variation of telomere length, including genetic and environmental factors^10–14^. These studies have shown that telomere length is a largely heritable trait, and is also affected by both environmental and genetic factors in a population-specific manner^22,23,26^. Due to involvement of the mentioned factors in telomere length variation, consistency of results across studies strongly depends on the study design, particularly on inclusion of families and multivariate analysis.

Here, we have analyzed whole genome sequencing data obtained by the genome of the Netherlands (GoNL) project, which included 250 Dutch family trios with associated meta-information on sex, age, paternal and maternal ages at conception. We have used computational means for estimation of telomere length and have performed a series of regression and quantitative trait association analyses to reveal inheritance patterns of telomeres and to identify genetic variations affecting their length.

While age plays a role in telomere length variation during a lifetime, our analysis show that its effect is weaker compared to inheritance factors, namely parental telomere lengths, with mother’s telomeres being the strongest predictors of offspring MTL. The observed highly heritable nature of telomeres is in agreement with prior studies performed on twins, showing that lymphocyte telomere length at birth largely determines inter-individual variation, while factors regulating telomeres during lifetime are of less importance^12,41^. However, whether length inheritance occurs stronger through paternal^27^ or, as in our case, through maternal line^28^, is still a controversy.

Our results also show positive association of maternal age at conception (MAC) with offspring MTL. Previous studies have largely linked paternal age at conception (PAC) with offspring TL^29^. This association has been explained by positive selection of sperm cells with longer telomeres with increasing age in males^27,30,31^. There is limited evidence on MAC effect on offspring TL due to high correlation between biological ages of partners and the difficulty to assess TL dynamics in healthy female oocytes^36^. Most studies perform regression analysis to support the hypothesis of PAC association with TL and monitor changes in PAC estimates before and after adjustment for MAC^40^. Our observation of positive association of MAC with offspring MTL was also confirmed after partial correlation analysis. It is a matter of future investigations to find out the causes for such a link: whether MAC directly affects TL in oocytes or is indirectly involved in TL regulation in the embryo.

All in all, in the studied population, offspring MTL is positively affected by maternal and paternal MTL and MAC. While these correlations could partially reflect mendelian inheritance of telomeres^32^, those could also depend on inheritance of genetic factors regulating TL. Identification of telomere-related genetic variants in our cohort was not trivial, due to multiple factors of TL variability that should be accounted for when performing such studies in families. In particular, family association tests implemented in Plink did not allow for inclusion of age as a confounder, while Merlin did not account for heritability of telomeres as quantitative traits. Furthermore, inclusion of genotypes for each SNV into our multiple linear regression model for TL inheritance did not account for family structure. These limitations, along with the small sample size, reduced the statistical power of our tests. Nevertheless, the only consistent hit – *RRM1* – was identified by Plink-based analysis, where age was the missing confounder. This once again confirmed the relatively small influence of age on TL variability among other factors.

*RRM1* encodes the catalytic subunit of ribonucleotide reductase (RNR), an enzyme essential for dNTP synthesis and DNA replication and repair processes^42^. In humans, the role of *RRM1* in telomere length regulation is not established yet. However, it is known that the yeast ortholog of this gene, *Rnr1*, is required for telomerase functionality, possibly via facilitation of telomere synthesis via local temporary boosts of dNTPs^43^. Our data, thus, suggests that *RRM1* may also be involved in telomere length regulation in humans. Importantly, it is known that in humans, *RRM1* is involved in regulation of cellular proliferation, migration and cancer progression, presumably due to its role in DNA replication^44^. It is also targeted by certain chemotherapeutics or used as a biomarker to predict their effectiveness^44–46^. Furthermore, polymorphisms and altered expression of this gene have been linked to increased susceptibility to certain cancers^45,47^. *RRM1*’s role in cancer, however, is only discussed in terms of its involvement in DNA synthesis, and not in terms of telomere length regulation. This study, thus, may serve as a basis for extending the functional studies on *RRM1* to telomeres as well.

## Conclusions

All in all, the well-structured and richly annotated dataset provided by the Genome of the Netherlands project has allowed us to reveal several aspects of telomere length inheritance and variability. We have shown that parents’ telomere lengths and mother’s age at conception play an important role in defining telomere length variability among individuals, along with age and genetic factors. Additionally, our results on association of *RRM1* with MTL and prior studies on its ortholog in yeast, suggest possible implications for this gene in telomere biology in humans.

We also highlight the limitations of currently available computational tools for performing genome-wide association studies on heritable quantitative traits in family trio datasets. Development of these tools and emergence of sequencing datasets on family trios from other populations will further allow for delineating common and population-specific factors involved in telomere length regulation.

## Methods

### Datasets

We make use of secondary analysis of whole genome sequencing (WGS) data (http://www.nlgenome.nl/, European Genome-phenom Archive, EGAS00001000644) previously generated by the Genome of the Netherlands Project (GoNL). The WGS data had been produced on Illumina HiSeq. 2000 platforms with 12x coverage. It consisted of 748 Dutch individuals forming trios of a mother, a father and a child. We also had information about their age and sex, as well as the ages of conception for the mothers and the fathers.

Single nucleotide variation (SNV) data was obtained from the release 5.4 of the GoNL project, where variant calling had been performed on hg19 assembly of the reference genome by the GoNL Consortium^33^.

### SNV filtering

We have accounted for SNV data passing the filters with VCFtools version 0.1.14, and have removed SNVs with minor allele frequency <5% (70.8% of all SNVs), those with missingness values of <10% (0.3% of all SNVs) and those not passing Hardy-Weinberg equilibrium significance threshold of 0.001 (1.2% of all SNVs) using Plink version 1.9. Overall, genotype information for 5352580 SNVs for 748 individuals were included in the downstream analyses.

### Telomere length calculation

We have calculated mean telomere lengths (MTL) from whole genome sequencing data using Computel version 0.4 with its default parameters^34^. Computel determines MTL by alignment to a special telomeric reference and by comparing the coverage at the telomeric reference to the coverage at the genome reference^34^. The WGS data contained multiple runs per individual, and we have taken the median MTL per individual across multiple runs.

### Regression analysis

Multivariate linear regression analysis was performed to evaluate the correlation between MTL and age and sex in the studied population. Another MLR model was analyzed to study the inheritance of maternal and paternal MTL, as well as the role of MAC and PAC on offspring MTL. Two of the families with missing data for the mother were removed, and two families with discordant age differences at the time of data collection and at conception were also discarded. Overall, MLR were done on 246 families. A set of pairwise regressions on the predictors were performed to estimate dependence between variables, and interaction terms were introduced for correlated predictors. The MLR models were tested by sequential introduction of predictors and interaction terms. The best model was chosen based on maximization of the adjusted R square term: ultimately, from the three best models with similar adjusted R squared values the simplest one was chosen. Partial correlation analyses were performed with R package ppcor and were used to estimate the influence of each predictor when accounting for the rest of the predictors in the model.

### Telomere qTL analysis

Association of single nucleotide variations (SNV) with MTL was performed with family based association tests for quantitative traits implemented in Plink 1.9^48^ and Merlin 1.1.2^49^, as well as with a series of MLR models. Both, Merlin and Plink use the between/within model implemented in the QTDT package^50^. However, Merlin uses a maximum likelihood variance component model to correct for family structure, while Plink uses a procedure based on permutations.

We ran Plink with the qfam-total option for identification of quantitative associations with adjustment for sex, and with 100 000 000 permutations to adjust for family structure. One drawback of this approach is that Plink does not handle covariates (other than sex) for family associations, which did not allow to adjust for age. As age was affecting telomere length in our analysis (see Results), we also ran a number of alternative tests.

Separate linear regression tests for parents and children were performed with Plink quantitative association tests, using an additive model for variants and treating age and sex as covariates.

To perform family association tests accounting for age, we used Merlin. For this, we have downloaded genetic maps of 1000 genomes project variants generated by Hapmap 2 project that involved average recombination rates in three different populations (CEU, YRI, and ASN) (from https://github.com/joepickrell/1000-genomes-genetic-maps). Only the coding SNVs were included in these tests, and those SNVs with mendelian errors were marked as missing. While Merlin accounted for age, it had the disadvantage of not accounting for quantitative trait inheritance, i.e. inheritance of telomere lengths from parents to offspring, as well as being limited to variants for which recombination rate information is available.

Finally, we also ran multiple linear regression (MLR) analysis by incorporating an additive model of child’s genotype for each variant (a variable taking on values from 0 to 2, according to the number of minor alleles in the genotype) into the MLR model of telomere length inheritance obtained from MLR analysis described above. While these regression models do not account for variant linkage and thus had lower statistical power, they do consider telomere length inheritance, age and family structure.

## Supplementary information

Supplementary document 1

Supplementary datasheet 1

Supplementary table S1

## Data Availability

This study makes use of the data generated by the Genome of the Netherlands Project. A full list of the investigators is available from www.nlgenome.nl. The datasets analyzed during the current study are available in the European Genome-phenome Archive repository, https://www.ebi.ac.uk/ega/studies/EGAS00001000644. The scripts used for the analyses are available in Github, at https://github.com/lilit-nersisyan/gonl_tqtl.
