# Supplementary material for: WGS-based telomere length analysis in Dutch family trios implicates stronger maternal inheritance and a role for RRM1 gene
Source: Sci Rep. 2019 Dec 10;9:18758. doi: 10.1038/s41598-019-55109-7 (PMC6904582; doi:10.1038/s41598-019-55109-7)
Supplement: Supplementary file 1 — Supplementary document 1 [file 41598_2019_55109_MOESM1_ESM.pdf]

Supplementary document 1

WGS-based telomere length analysis in Dutch family trios implicates stronger maternal inheritance and a role for RRM1 gene

Lilit Nersisyan[*,1], Maria Nikoghosyan[1,2], Genome of the Netherlands Project[3], Arsen Arakelyan[1,2]

1. Bioinformatics Group, Institute of Molecular Biology NAS RA, 7 Hasratyan str., 0014, Yerevan, Armenia
2. Institute of Biomedicine and Pharmacy, Russian-Armenian University, 123 Hovsep Emin St, 0051, Yerevan, Armenia
3. Genome of the Netherlands Project Consortium

*Correspondence: Lilit Nersisyan, l_nersisyan@mb.sci.am, Bioinformatics Group, Institute of Molecular Biology NAS RA, 7 Hasratyan str., 0014, Yerevan, Armenia

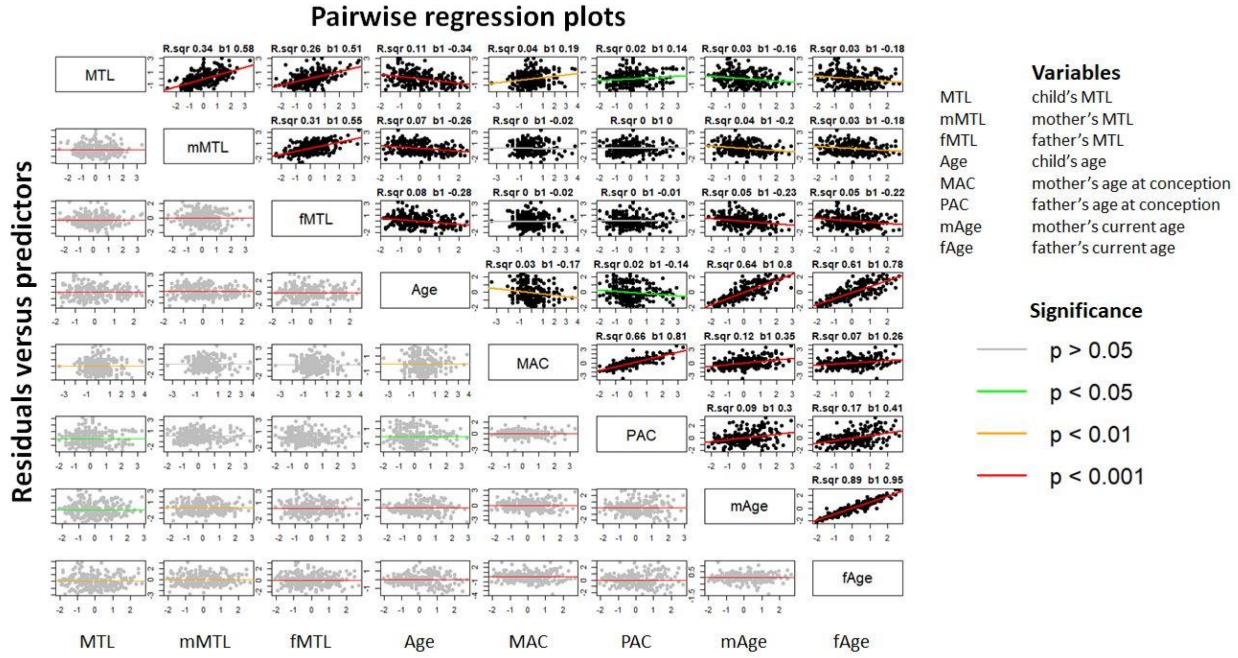

Figure S1. Pairwise correlations between offspring mean telomere length (MTL) and meta-variables.

The right triangle shows correlation plots with fitted correlation lines and linear regression statistics (R squared and correlation coefficient). The left triangle shows residuals of each correlation with respect to the predictive variable. We see that mother's and father's ages, as well as PAC and MAC show strong correlation, which should be considered in multiple linear regression analysis (see the main text for details).
